# A thalamo-cortical model to explain EEG during anaesthesia

**DOI:** 10.1186/1471-2202-14-S1-P177

**Published:** 2013-07-08

**Authors:** Meysam Hashemi, Axel Hutt

**Affiliations:** 1 INRIA CR Nancy - Grand Est, 54600 Villers-les-Nancy Cedex, France

## 

General anesthesia (GA) is a neurophysiological state which includes analgesia, amnesia, immobility and skeletal muscle relaxation. Although GA is commonly used in medical care for patients undergoing surgery, its precise underlying mechanisms and the molecular action of anesthetic agents (AA) remain to be elucidated. A wide variety of drugs are used in modern anesthetic practice and it has been observed that for most AAs, during the transition from consciousness to unconsciousness, many derived EEG variables show biphasic effects, that is an initial increase of the effect variable followed by a decrease at higher concentrations. Moreover during the administration of propofol, specific changes in EEG rhythms can be observed.

The aim of our work is to describe mathematically this bi-phasic behavior in the EEG-power spectrum [1] and reproduce changes in EEG rhythms [2] by the study of a neuronal population model of a single thalamo-cortical module [3]. The model distinguishes excitatory and inhibitory synapses and considers the effect of propofol on GABAergic synaptic receptors in thalamic cells. The work finds a power peak in the alpha-range that moves to higher frequencies with increasing propofol concentration, while activity in the delta-frequency range increases as well. It is important to point out that the model neglects the propofol effect in cortical cells and considers just thalamic molecular action in contrast to previous studies, e.g. [2]. This indicates the importance of the thalamus and weakens the impact of the cortex for major neural effects under anaesthesia.
